# Wavelength-Specific Transcriptomic Responses of *Diaphorina citri* to Nocturnal Monochromatic LED Light

**DOI:** 10.3390/insects17070735

**Published:** 2026-07-17

**Authors:** Shuai Xu, Zhihang Chen, Mohao Xiong, Jiangwen Huang, Huixin Wang, Bin Wan, Guangxu Wang, Bin Xia

**Affiliations:** 1 School of Life Sciences, Nanchang University, Nanchang 330031, China; xushuai@email.ncu.edu.cn (S.X.); czhang2826@163.com (Z.C.); xmh0804y@126.com (M.X.);; 2 National Institute of LED on Si Substrate, Nanchang University, Nanchang 330047, China

**Keywords:** artificial light at night, *Diaphorina citri*, opsin, kynurenine pathway, lysosome, autophagy

## Abstract

The proliferation of light-emitting diodes (LEDs) in urban and agricultural settings has intensified artificial light at night (ALAN), yet the molecular mechanisms underlying its spectrally specific perturbation of insect physiology remain elusive. We used high-throughput RNA sequencing to dissect the transcriptomic responses of *Diaphorina citri*—a principal vector of citrus Huanglongbing—to nocturnal exposure to monochromatic LED emissions at 460 nm (blue), 520 nm (green), 570 nm (yellow), and 620 nm (red). Blue light triggered metabolic reprogramming coupled with stress responses. Red light predominantly engaged lysosomal–autophagic pathways and lipid metabolism. Yellow and green light produced only modest transcriptional alterations with no significant pathway enrichment. These results demonstrate that *D. citri* exhibits a markedly wavelength-dependent transcriptomic response to nocturnal monochromatic illumination, with short-wavelength blue and long-wavelength red light eliciting pronounced physiological effects through distinct molecular routes, thereby providing mechanistic insight into wavelength-specific ALAN impacts and a rationale for targeted optical pest management strategies.

## Simple Summary

The rapid expansion of LED lighting has intensified artificial light at night (ALAN) pollution, but the molecular impacts of different wavelengths on insects remain poorly understood. Using RNA sequencing, we investigated the transcriptomic responses of *Diaphorina citri*—the principal vector of citrus greening disease—to nocturnal exposure to blue, red, yellow, and green LED light. Blue and red light elicited the most pronounced gene expression changes: blue light triggered metabolic reprogramming coupled with stress responses, whereas red light activated the lysosome–autophagy pathway. In contrast, yellow and green light induced only modest transcriptional alterations. These wavelength-specific molecular patterns advance our understanding of ALAN’s biological consequences and provide a mechanistic foundation for spectrally targeted pest management.

## 1. Introduction

The rapid development and widespread application of light-emitting diode (LED) lighting technology have profoundly transformed human production and lifestyle patterns. LED light sources, characterized by high energy efficiency, long lifespan, and adjustable spectral properties, have gradually become the mainstream for urban nighttime illumination [1,2,3]. However, the escalating problem of light pollution arising from nighttime lighting projects and improper illumination exerts substantial negative impacts on the biosphere. Light sources of different wavelengths exert differential effects on organisms. Specifically, short-wavelength blue light (400–470 nm) can penetrate biological tissues, inducing cellular-level oxidative stress responses, protein denaturation, and DNA damage [4,5]. Medium-wavelength green light (500–560 nm) and yellow light (560–590 nm) primarily affect organismal behavior through the disruption of biological rhythms and visual systems [6,7,8,9]. Long-wavelength red light (600–750 nm), although possessing lower energy, can still influence endocrine systems and circadian rhythms [10,11,12]. Light radiation across different wavebands produces specific biological effects on organisms by activating particular photoreceptors and triggering differentiated physiological and biochemical cascade reactions [13].

Insects, representing the most species-rich animal group on Earth, fulfill critical ecological functions in ecosystems, including pollination, natural enemy control, nutrient cycling, and energy transfer through food webs [8,14]. Nevertheless, artificial light at night (ALAN) poses severe threats to insect populations [15]. Numerous studies demonstrate that ALAN interferes with normal insect biological functions through multiple mechanisms [16,17]. At the behavioral level, phototactic responses of insects toward light sources increase energy expenditure and predation risk, while insects that rely on light for orientation become disoriented due to ALAN [18,19]. At the physiological level, nighttime illumination inhibits the synthesis and release of sex pheromones, significantly reducing mating success rates [20,21,22]. At the population level, insect populations exposed to ALAN over extended periods also experience significant impacts [23,24,25].

*Diaphorina citri* Kuwayama serves as the specialized vector for Huanglongbing (HLB), a disease caused by the phloem-restricted bacterium *Candidatus Liberibacter asiaticus* (CLas) and representing one of the most devastating threats to the citrus industry [26,27]. The feeding behavior of *D. citri* not only causes direct damage to citrus plants but, more critically, leads to substantial yield reduction and quality decline through the transmission of HLB pathogens, resulting in enormous economic losses [28]. In recent years, researchers have begun to investigate the associations between light environmental factors and the biological characteristics of *D. citri*. Studies indicate that *D. citri* exhibits differential phototactic behavioral responses to various light wavebands, such as strong positive phototaxis toward ultraviolet (350–405 nm) and violet (400–430 nm) light, while showing no significant differences in response to green (500 nm), yellow (580 nm), and orange-red (620 nm) light compared to dark environments [29]. Additional research reveals that blue light (400 nm) can activate photoreceptors in the head of *D. citri*, triggering differential expression of genes related to oxidative stress, protein denaturation, and neuromodulation, thereby converting light signals into electrical signals and regulating phototactic behavior [30]. Furthermore, polarized light and light intensity are also demonstrated to significantly affect walking distance and orientation behavior in *D. citri* [29]. Although the proliferation of LED light sources in plantations and related agricultural areas has markedly enhanced agricultural productivity, the resultant effects on agricultural pests have received limited research attention. Given the spectral sensitivity of insect photoreceptors and the documented physiological impacts of short-wavelength light on metabolic homeostasis, we hypothesized that nocturnal monochromatic LED irradiation exerts wavelength-specific transcriptional reprogramming in *D. citri*, whereby discrete wavelengths elicit divergent metabolic and physiological responses. To test this hypothesis, we employed high-throughput RNA sequencing (RNA-seq) to systematically analyze the transcriptome of *D. citri* subjected to nighttime illumination treatment with four LED wavelengths (blue: 460 nm, green: 520 nm, yellow: 570 nm, and red: 620 nm). Our objective is to elucidate the effects of continuous nighttime monochromatic light factors on *D. citri*.

## 2. Materials and Methods

### 2.1. Insects

The *D. citri* specimens utilized in this study were derived from a laboratory colony maintained via successive rearing for over five years on healthy Murraya paniculata plants without exposure to CLas-infected citrus material. The founding population was initially collected from the field and subsequently established on the host plant *Murraya paniculata* under controlled laboratory conditions. Rearing parameters were standardized as follows: temperature at 25 ± 2 °C, relative humidity at 75 ± 5%, and a photoperiod of 14:10 h (light:dark). Throughout the rearing process, populations were housed within mesh cages (60 cm × 60 cm × 90 cm). Strict protocols were implemented to prevent exposure to pesticides and any extraneous light contamination outside of experimental treatments. Furthermore, healthy host plants were replaced periodically to ensure the viability and quality of the insect colony. Given that both sexes are equally exposed to nocturnal ALAN under field conditions, mixed-sex cohorts were considered representative of ecologically realistic scenarios. Accordingly, each experimental group comprised adult individuals of both sexes without prior sex separation.

### 2.2. Reaction Chamber Setup

**Figure 1:**
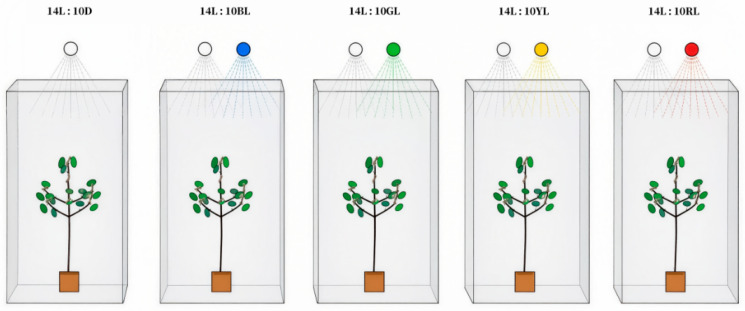
Schematic diagram of the experimental setup.

The experiment was conducted using five mesh cages as experimental units, each enclosed by black opaque curtains to eliminate extraneous light interference. White LED sources were installed uniformly atop all five cages to simulate diurnal illumination. Four of these cages were additionally equipped with monochromatic LED arrays emitting blue (B: 460 nm), green (G: 520 nm), yellow (Y: 570 nm), or red (R: 620 nm) light, respectively, for nighttime treatment, whereas the fifth cage was maintained in complete darkness during the night period as the control. Each light source was operated via an independent, dimmable circuit. Light intensity was calibrated by adjusting the power output to achieve 600 lux for white light and 80 lux for each monochromatic wavelength, measured at the central bottom position of the cages using a portable spectrometer (Model PLA-30; Hangzhou Everfine Photo-e-info Co., Ltd., Hangzhou, China). The control group was subjected to a standard photoperiod of 14L:10D (light:dark). In contrast, the experimental groups were exposed to a 14L:10ML regime, consisting of 14 h of white light followed by 10 h of monochromatic light irradiation daily. The experimental setup is schematically illustrated in Figure 1.

### 2.3. Sample Collection, RNA Extraction, and Sample Quality Control

Total RNA was extracted from healthy citrus psyllids (five days post-eclosion) collected two hours after the photoperiod switch from L to D or ML. Samples were flash-frozen in liquid nitrogen prior to extraction using TRIzol reagent (Sangon Biotech, Shanghai, China). RNA integrity was assessed using the RNA Nano 6000 Assay Kit of the Bioanalyzer 2100 system (Agilent Technologies, Santa Clara, CA, USA) [30].

### 2.4. Library Preparation and Transcriptome Sequencing (RNA-seq)

Total RNA was used as input material for the RNA sample preparations. Briefly, mRNA was purified from total RNA using poly-T oligo-attached magnetic beads. Fragmentation was carried out using divalent cations under elevated temperature in First Strand Synthesis Reaction Buffer (5X). First-strand cDNA was synthesized using random hexamer primers and M-MuLV Reverse Transcriptase (RNase H-). Second-strand cDNA synthesis was subsequently performed using DNA Polymerase I and RNase H. Remaining overhangs were converted into blunt ends via exonuclease/polymerase activities. After adenylation of the 3’ ends of DNA fragments, adaptors with a hairpin loop structure were ligated to prepare for hybridization. In order to select cDNA fragments of preferentially 370~420 bp in length, the library fragments were purified with the AMPure XP system (Beckman Coulter, Brea, CA, USA). Then, PCR was performed with Phusion High-Fidelity DNA polymerase, Universal PCR primers, and Index (X) Primer. At last, PCR products were purified (AMPure XP system), and library quality was assessed on the Agilent Bioanalyzer 2100 system [31,32].

The clustering of the index-coded samples was performed on a cBot Cluster Generation System using TruSeq PE Cluster Kit v3-cBot-HS (Illumina, San Diego, CA, USA) according to the manufacturer’s instructions. After cluster generation, the library preparations were sequenced on an Illumina Novaseq platform, and 150 bp paired-end reads were generated.

### 2.5. Bioinformatics Analysis

FeatureCounts v1.5.0-p3 was used to count the read counts mapped to each gene, and then the FPKM of each gene was calculated based on the length of the gene and the read count mapped to this gene. The data were then aligned to a reference genome (ncbi_diaphorina_citri_gca_024506315_2_crf_ca_dcit). Differential expression analysis (DEGs) of two conditions/groups (three biological replicates per condition) was performed using the DESeq2 R package (1.20.0). DESeq2 provides statistical routines for determining differential expression in digital gene expression data using a model based on the negative binomial distribution. The resulting *p*-values were adjusted using the Benjamini–Hochberg approach for controlling the false discovery rate. DEGs were filtered using the following criteria: |log2 fold change(FC)| > 1 and padj < 0.05 [33].

Gene Ontology (GO) enrichment analysis of differentially expressed genes was implemented by the clusterProfiler R package, in which gene length bias was corrected. GO terms with corrected padj less than 0.05 were considered significantly enriched by differentially expressed genes [34]. The Kyoto Encyclopedia of Genes and Genomes (KEGG) is a database resource for understanding high-level functions and utilities of the biological system, such as the cell, the organism, and the ecosystem, from molecular-level information, especially large-scale molecular datasets generated by genome sequencing and other high-throughput experimental technologies. We used the clusterProfiler R package to test the statistical enrichment of differentially expressed genes in KEGG pathways [35].

The raw transcriptome sequencing data generated in this study have been deposited in the NCBI Sequence Read Archive (SRA) under BioProject accession number PRJNA1492229. All other data supporting the findings of this study are available within the paper and its Supplementary Information.

### 2.6. Validation by RT-qPCR

To validate the accuracy of the digital gene expression (DGE) analysis, 16 genes were randomly selected. Primers were designed using Oligo 7 software, and the sequences for all genes are listed in Table S1. *β-Actin* (GenBank: DO675553) was used as the internal reference gene. RT-qPCR was performed using a StepOnePlus™ Real-Time PCR System (Thermo Fisher Scientific, Singapore), with each reaction conducted in triplicate. The relative expression levels of the genes were calculated using the 2^−ΔΔCt^ method [36].

## 3. Results

### 3.1. Summary of Transcriptome Sequencing and Assembly

Total RNA was extracted from the samples of the CK (control check), B, G, Y, and R groups. The transcriptome sequencing and assembly were generated by RNA sequencing. Fifteen cDNA samples, three replicates for each group, were sequenced by the Illumina Novaseq platform, with paired-end 150 nt reads. A total of 702,475,844 (ranging from 41096586 to 50223406) raw reads were obtained. After removing reads containing adapters, poly-N, and low-quality reads, we obtained 38.6–48.2 million clean reads with high-quality values (Q20 > 98.9%, Q30 > 96.4%, error rate = 0.01%), and GC content ranged from 35.79% to 45.2% following a normal distribution. Clean data were aligned to the reference genome via Hisat2 (v2.0.1); a total of 76.26–81.95% of the distinct reads could be mapped to the reference genome, and the clean reads could be uniquely mapped with values of 72.23–74.65%. The results are presented in Table 1.

### 3.2. Identification and Functional Analysis of Differentially Expressed Genes (DEGs)

**Figure 2:**
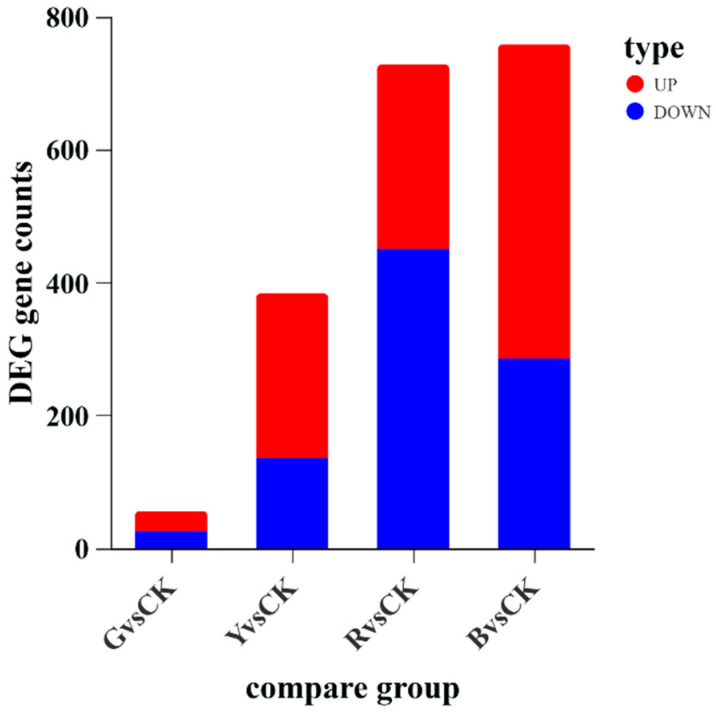
Statistics of differentially expressed genes (DEGs) in different light treatment groups compared with the control group.

**Figure 3:**
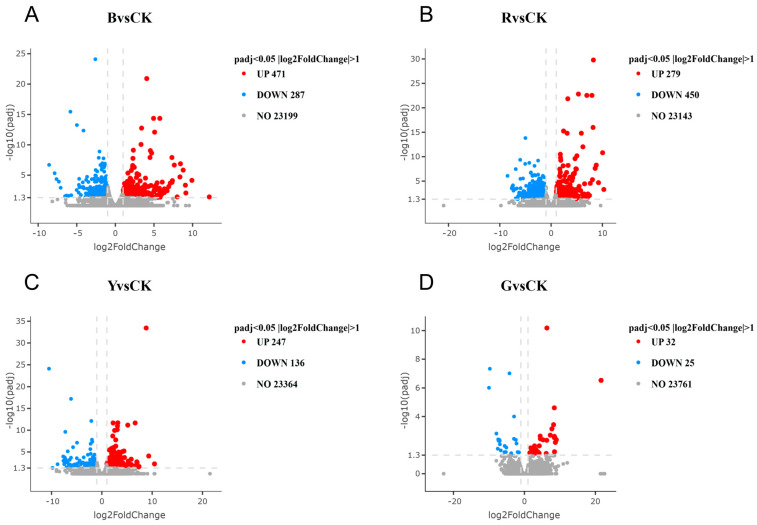
Volcano plots of differentially expressed genes in different light treatment groups compared with the control group. (**A**–**D**) Volcano plots comparing blue light (B), red light (R), yellow light (Y), and green light (G) treatment groups with the control check (CK), respectively. The x-axis indicates the fold change in gene expression (log2FoldChange), and the y-axis indicates the statistical significance (−log10(padj)). Red dots represent significantly up-regulated differentially expressed genes (padj < 0.05, |log2FoldChange| > 1), blue dots represent significantly down-regulated differentially expressed genes, and gray dots represent genes with no significant difference. The number of significantly differentially expressed genes in each treatment group is labeled in the plots.

Differential expression analysis revealed that a substantial number of differentially expressed genes (DEGs) were detected in all four light treatment groups compared with the control group (CK) (Figure 2). The volcano plots of differentially expressed genes are shown in Figure 3. The blue light treatment group (BvsCK) exhibited the highest number of DEGs, with 758 DEGs identified, including 471 up-regulated and 287 down-regulated genes. The red light treatment group (RvsCK) ranked second, with 729 DEGs in total, among which down-regulated genes (450) outnumbered up-regulated genes (279). The yellow light treatment group (YvsCK) displayed 383 DEGs, with up-regulated genes (247) significantly outnumbering down-regulated genes (136). In contrast, the green light treatment group (GvsCK) showed the weakest transcriptional response, with only 57 DEGs detected and predominantly up-regulated.

**Figure 4:**
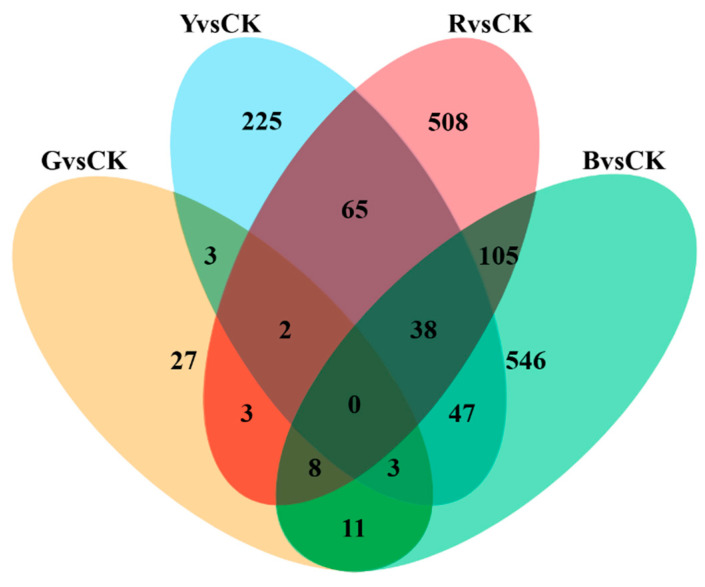
Venn diagram of differentially expressed genes among different monochromatic light treatment groups.

The Venn diagram illustrates the distribution and overlap of DEGs identified in all four light treatment groups compared with the CK (Figure 4). The numbers of group-specific DEGs are 546 for blue light, 508 for red light, 225 for yellow light, and 27 for green light. No DEGs were shared among all four treatment groups, indicating a pronounced spectral specificity in the transcriptional responses induced by different wavelengths of nocturnal illumination.

The detailed analysis of DEGs in BvsCK is presented in Table S2. Metabolic reprogramming-related genes were significantly up-regulated, among which glyoxylate reductase/hydroxypyruvate reductase (*GRHPR*) showed the most significant up-regulation. Kynurenine aminotransferase (*KAT*) was also among the top 10 significantly up-regulated DEGs, together with sorbitol dehydrogenase (*DHSO*) and aspartate aminotransferase (*AATC*). Immune defense-related protease genes were significantly up-regulated, including CLIP-domain serine proteases, viral cathepsins, and pro-cathepsin H (*CATH*). Among the significantly down-regulated DEGs, the β/γ-crystallin family member gene associated with vision showed the most prominent down-regulation. Additionally, three rhodopsin family seven-transmembrane receptor opsins were significantly down-regulated, namely, blue-sensitive opsin (*OPSB*), rhodopsin (*OPSD*), and ultraviolet-sensitive opsin (*OPSUV*). Furthermore, transposon-derived reverse transcriptase genes were significantly down-regulated, including two RNA-directed DNA polymerases from transposon BS (*RTBS*) and RNase H (*RNASEH*), all of which ranked among the top 10 most significantly down-regulated DEGs. These DEGs indicate that blue light suppressed vision-related proteins (crystallins and opsins), enhanced expression of metabolic reprogramming-related enzymes (kynurenine pathway and glyoxylate metabolism), and immune defense-related proteases (cathepsins and serine proteases), while retrotransposon activity was significantly inhibited.

The detailed analysis of DEGs in RvsCK is presented in Table S3. Among the significantly up-regulated DEGs, inclusion body protein showed the most significant up-regulation. Cathepsin B-like cysteine proteinase genes were markedly up-regulated, with both *CYSP1* and *CPR4* ranking among the top 10 significantly up-regulated DEGs. In addition, lipid metabolism-related genes, such as putative fatty acyl-CoA reductase (*FACR1*) and vitellogenin-2 (*VIT2*), were significantly up-regulated; juvenile hormone esterase (*JHE*) and lysosomal acid glucosylceramidase (*GLCM*) were also among the top 10 significantly up-regulated DEGs. Other significantly up-regulated genes included excitatory amino acid transporter 3 (*EAA3*), glucose dehydrogenase (*DHGL*), and bifunctional glutamate/proline--tRNA ligase (*SYEP*). Among the significantly down-regulated DEGs, basic phospholipase A2 (*PA2B5*) showed the most significant down-regulation, being the only down-regulated gene among the top 10 DEGs. Additionally, insect cuticle protein and hemolymph juvenile hormone binding protein (*JHBP*) were significantly down-regulated. Among vision-related genes, rhodopsin (*OPSD*) and β/γ-crystallin gene were down-regulated. Furthermore, immune-related genes, such as CD80-like C2-set immunoglobulin domain and peroxidasin homolog pxn-2 (*PXDN2*), were significantly down-regulated. Metabolic enzyme genes including glutaminase kidney isoform (*GLSK*), gastric triacylglycerol lipase (*LIPG*), and vacuolar (H+)-ATPase G subunit were also significantly down-regulated. These DEGs indicate that red light altered protease gene expression of protease activity-related genes and enhanced expression of lipid metabolism-related genes, while vision-related proteins and insect cuticle structural proteins were suppressed, and hormone signaling-related proteins were down-regulated.

The detailed analysis of DEGs in YvsCK is presented in Table S4. Among the significantly up-regulated DEGs, inclusion body protein showed the most significant up-regulation. Glycogen debranching enzyme (*GDE*), excitatory amino acid transporter 3 (*EAA3*), viral cathepsin (*CATV*), and galactose binding lectin domain also ranked among the top 10 significantly up-regulated DEGs. Other significantly up-regulated genes included formin-like protein (*FRL*), spectrin alpha chain (*SPTCA*), and Ras-related protein Rab-30 (*RAB30*). Among the significantly down-regulated DEGs, V-type proton ATPase subunit G (*VATG*) showed relatively prominent down-regulation. Vision-related genes rhodopsin (*OPSD*) and blue-sensitive opsin (*OPSB*) both ranked among the top 10 significantly down-regulated DEGs. Poly ADP-ribose glycohydrolase (*PARG*), follistatin (*FST*), and testicular acid phosphatase homolog (*PPAT*) were also significantly down-regulated. In addition, multiple reverse transcriptases (RNA-dependent DNA polymerases) showed significant down-regulation. These DEGs indicate that yellow light treatment resulted in significant up-regulation of inclusion body protein genes, altered expression of protease activity-related genes, and enhanced expression of carbohydrate metabolism and lipid metabolism-related genes; meanwhile, vision-related protein genes were down-regulated, ATPase-related genes were suppressed, and hormone signaling-related proteins and transposon elements exhibited significant down-regulation.

The detailed analysis of DEGs in GvsCK is presented in Table S5. Among the significantly up-regulated genes, metabolic enzymes, such as fatty acyl-CoA reductase (*FACR1*) and alkaline phosphatase (*PPBT*), showed significantly enhanced expression; immune defense-related proteases including pro-cathepsin H (*CATH*), CLIP domain-containing serine protease 2, and melanization protease 1 (*MP1*) were significantly up-regulated. Among the significantly down-regulated genes, allergen Cr-PI (*CRPI*) showed the most significant down-regulation. Transposon-related genes, such as reverse transcriptase and LINE-1 retrotransposable element ORF2 protein (*LORF2*), were significantly down-regulated. In addition, transport and oxidation-related proteins, including peroxidasin homolog pxn-2 (*PXDN2*) and sodium/hydrogen exchanger 8 (*Q8R4D1/SL9A8*), were suppressed. These DEGs indicate that green light treatment resulted in enhanced expression of metabolic enzymes and immune defense proteases, while transposon activity-related genes and ion transport proteins were inhibited.

### 3.3. KEGG and GO Enrichment Analysis of DEGs

**Figure 5:**
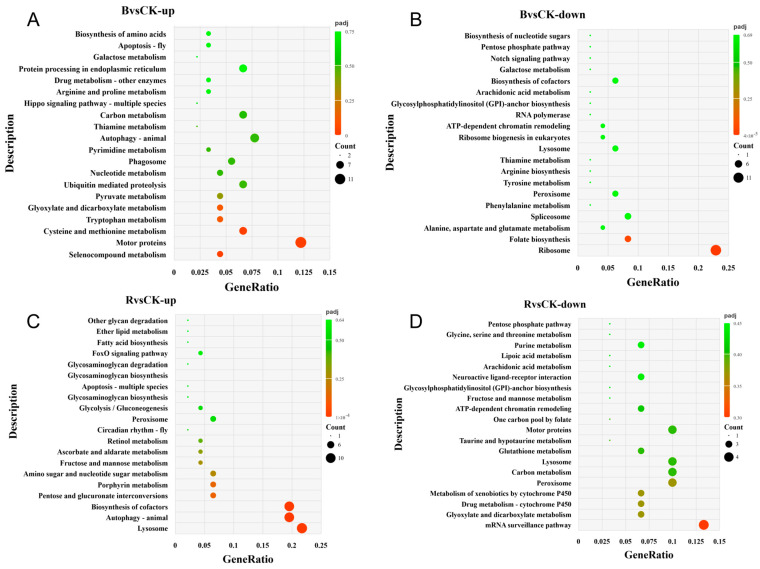
Bubble diagram of top twenty KEGG pathway enrichments up-regulated (**A**,**C**) and down-regulated (**B**,**D**) in BvsCK (**A**,**B**) and RvsCK (**C**,**D**) comparisons, respectively.

In the KEGG database, five pathways were significantly enriched in BvsCK (Table S6, padj < 0.05). Pathway analysis revealed that selenocompound metabolism, motor proteins, and cysteine and methionine metabolism were significantly up-regulated (Figure 5A), whereas ribosome and folate biosynthesis were significantly down-regulated (Figure 5B). For RvsCK, only three pathways showed significant enrichment (Table S7), all of which were up-regulated: lysosome, autophagy–animal, and biosynthesis of cofactors (Figure 5C,D). Notably, no significantly enriched pathways were detected for either GvsCK or YvsCK in the KEGG database, suggesting that these treatments induced limited transcriptional reprogramming or that the responses were not enriched in defined KEGG pathways under the current experimental conditions (Tables S8 and S9).

**Figure 6:**
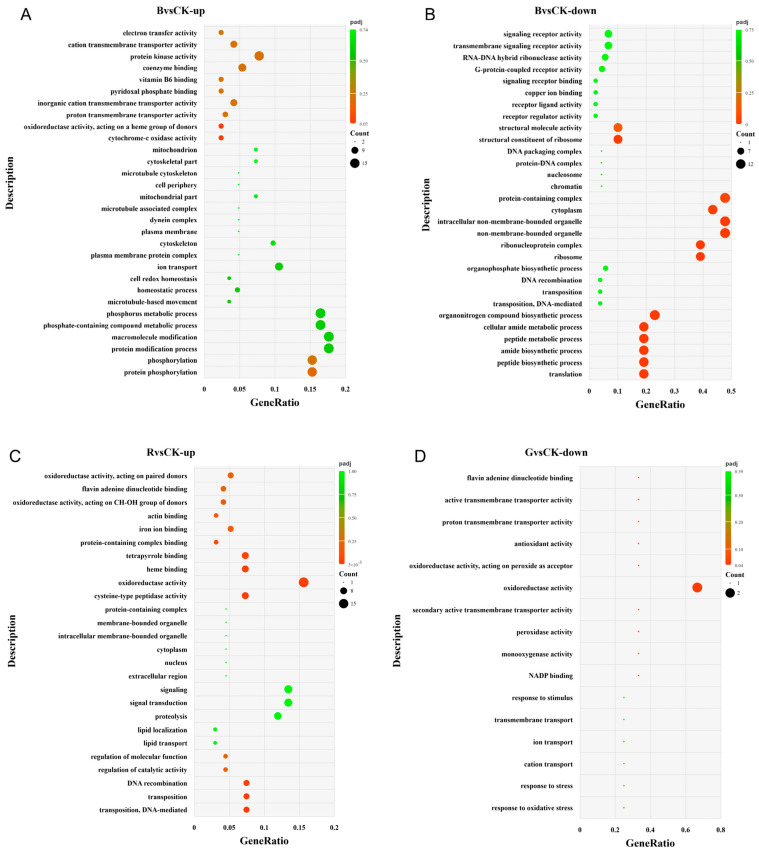
Bubble diagram of GO term enrichments. (**A**) Thirty up-regulated GO terms in BvsCK; (**B**) thirty down-regulated GO terms in BvsCK; (**C**) twenty-six up-regulated GO terms in RvsCK; and (**D**) sixteen down-regulated GO terms in GvsCK.

In the GO database, BvsCK exhibited significant enrichment of two up-regulated terms and 13 down-regulated terms (Figure 6A,B, Table S10). Among these, six terms belonged to biological process (BP), six to cellular component (CC), and three to molecular function (MF). Within the down-regulated terms, translation, peptide biosynthetic process, amide biosynthetic process, peptide metabolic process, cellular amide metabolic process, and organonitrogen compound biosynthetic process represented the most significantly enriched BP terms; ribosome, ribonucleoprotein complex, non-membrane-bounded organelle, and intracellular non-membrane-bounded organelle were significantly enriched in CC; and structural constituent of ribosome was significantly enriched in MF. In the up-regulated terms, cytochrome-c oxidase activity and oxidoreductase activity acting on a heme group of donors showed the most significant MF enrichment. The RvsCK only exhibited significant enrichment of seven up-regulated terms with no significantly down-regulated terms detected (Figure 6C, Table S11). Among these, three terms belonged to BP and four to MF. Within the up-regulated terms, transposition DNA-mediated, transposition, and DNA recombination represented the most significantly enriched BP terms, whereas cysteine-type peptidase activity, oxidoreductase activity, heme binding, and tetrapyrrole binding were significantly enriched in MF. GvsCK only exhibited significant enrichment of seven down-regulated terms with no significantly up-regulated terms detected (Figure 6D, Table S12). All significant terms belonged to the MF category. The most significantly enriched MF terms included NADP binding, monooxygenase activity, peroxidase activity, secondary active transmembrane transporter activity, oxidoreductase activity, oxidoreductase activity acting on peroxide as acceptor, and antioxidant activity. Notably, no significantly enriched GO terms were detected in YvsCK (Table S13). This suggests that the gene expression regulatory pattern under yellow light treatment conditions may not have induced significant GO pathway enrichment changes.

### 3.4. RT-qPCR Validation Study

**Figure 7:**
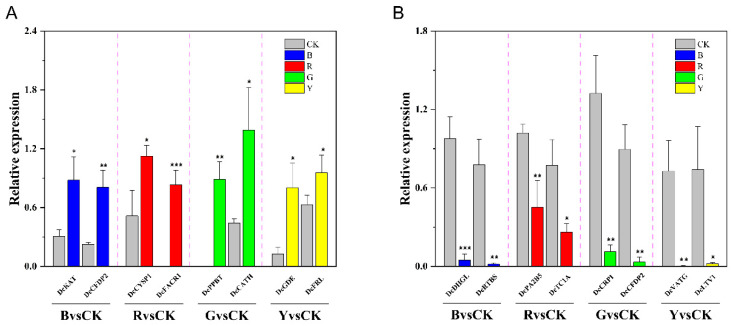
Transcriptome validation. (**A**) Validation of eight up-regulated genes in the four light-treated groups; (**B**) validation of eight down-regulated genes in the four light-treated groups by RT-qPCR. Student’s *t*-test was used for the data amalysis. Asterisks indicate significant differences (*t*-test; * *p* < 0.05,** *p* < 0.01, *** *p* < 0.001).

The accuracy of the DGE analysis was verified by RT-qPCR. Sixteen genes were randomly selected, including up-regulated and down-regulated genes from four treatment groups. The RT-qPCR results showed similar expression patterns as those of the DGE analysis (Figure 7). Therefore, the DGE approach was reliable.

## 4. Discussion

Despite the increasing prevalence of LED lighting in agricultural production, attributed to its unique advantages of energy conservation and spectral tunability, the biological effects on agricultural pests have yet to receive adequate research attention. Building upon the observed wavelength-dependent DEG gradients (Figure 2, Figure 3 and Figure 4; Tables S2–S5), these findings suggest that *D. citri* exhibits distinct wavelength-selective molecular responses to light, with short-wavelength blue light and long-wavelength red light inducing the most pronounced transcriptome reprogramming, while mid-wavelength green and yellow light produced relatively milder biological effects.

This discovery is highly consistent with the spectral sensitivity characteristics observed in behavioral studies of *D. citri*. *D. citri* exhibits strong positive phototaxis to UV/blue light but minimal response to mid- and long-wavelength light (500–620 nm) [29,37]. From a mechanistic perspective, this differential response likely originates from the distribution characteristics of photoreceptors in the insect visual system. Li et al. investigated the functions of opsin genes in *D. citri* in detail [38]. In the nocturnal moth *Ectropis grisescens* Warren, exposure to monochromatic light triggers rapid light adaptation, with opsin gene expression exhibiting significant diurnal plasticity under different light regimes [39]. The hierarchical opsin suppression pattern we observed—comprehensive down-regulation under blue light, selective suppression under yellow and red light, minimal effect under green light—parallels the quantitative gradient of transcriptome-wide responses. Notably, although red light treatment resulted in significant down-regulation of only one opsin gene, a substantial number of DEGs (729) were detected, suggesting that red light may exert physiological effects on *D. citri* through non-visual pathways, such as circadian rhythm regulation, endocrine disruption, or metabolic reprogramming. These results provide transcriptomic insights into the adaptive regulatory strategies of *D. citri* photoreceptor system at the molecular level, potentially informing our understanding of its spectral sensitivity and the physiological costs associated with phototactic behavior. Direct physiological validation would be required to confirm these inferred molecular consequences.

From an evolutionary ecology perspective, this wavelength-specific transcriptome response pattern reflects the long-term adaptation of *D. citri* to natural light environments. Under natural conditions, nighttime environments are dominated by long-wavelength red and near-infrared light, whereas short-wavelength blue light is primarily present during daytime [40,41,42]. Therefore, nighttime exposure to blue light represents an unnatural environmental signal that may activate stress response pathways in insects; conversely, although red light is a natural spectral component of nighttime, continuous illumination from artificial sources may interfere with photoperiod-dependent physiological rhythms. In this study, the green light treatment group exhibited a weak transcriptome response with only 57 DEGs detected, which may stem from co-evolution with the green background coloration of host plants.

Notably, the down-regulation of crystallin proteins, which function as structural components of the insect eye lens and also exhibit chaperone-like protective activities, was particularly marked under blue light. β/γ-crystallins represent the most significantly down-regulated DEG in the BvsCK comparison. Given that crystallins have been implicated in protecting ocular tissues from UV-induced damage and oxidative stress in various organisms [43], their suppression at the transcript level is consistent with a hypothetical trade-off where blue light-mediated phototoxicity may compromise the protective capacity of the visual system, pending direct experimental confirmation. This is consistent with the observation that blue light acts as an abiotic stressor in *D. citri*, triggering physiological responses, including oxidative stress and protein denaturation [31].

The most striking transcriptional response to blue light exposure involved extensive metabolic reprogramming, characterized by the up-regulation of genes associated with amino acid catabolism and cellular redox modulation. Among the top up-regulated DEGs, glyoxylate reductase/hydroxypyruvate reductase (*GRHPR*) and kynurenine aminotransferase (*KAT*) exhibited exceptionally high fold changes. The encoded GRHPR protein catalyzes the NADH-dependent reduction of glyoxylate to glycolate, a critical reaction that prevents glyoxylate accumulation and subsequent oxidative damage [44]. The marked induction of *GRHPR* transcript is suggestive of potential enhanced flux through the glyoxylate metabolic pathway, which may represent a putative protective mechanism against blue light-induced oxidative stress. KEGG enrichment analysis is consistent with significant up-regulation of the glyoxylate and dicarboxylate metabolism pathway in BvsCK.

The profound up-regulation of *KAT*, which catalyzes the transamination of kynurenine to kynurenic acid, implicates activation of the kynurenine pathway (KP) of tryptophan metabolism. The KP serves as a major route for tryptophan catabolism and produces bioactive metabolites with diverse immunomodulatory and neuroactive properties [45]. Kynurenic acid functions as an antagonist of NMDA-sensitive glutamate receptors and exhibits anti-inflammatory and immunosuppressive activities through aryl hydrocarbon receptor (AhR) signaling [46,47]. In insect systems, xanthurenic acid, a downstream KP metabolite generated by *KAT* activity, has been detected in the gut of *Aedes aegypti* Linnaeus mosquitoes, where it may exert antioxidant effects by binding heme and iron chelators [48]. The observed increase in *KAT* expression hints at a possible engagement of the kynurenine pathway’s neuroprotective branch by blue light, potentially representing a compensatory mechanism to counteract excitotoxicity and inflammatory damage linked to high-energy visible light.

The KEGG pathway analysis also revealed significant enrichment of selenocompound metabolism and cysteine and methionine metabolism among up-regulated pathways under blue light. Selenoproteins and sulfur-containing amino acid metabolites play fundamental roles in antioxidant defense and redox homeostasis maintenance [49]. The concurrent enrichment of these terms, coupled with the observed up-regulation of cytochrome-c oxidase activity, is suggestive of a coordinated cellular response that may operate to mitigate blue light-induced reactive oxygen species (ROS) generation. Previous studies have established that blue light irradiation stimulates ROS production in insect cells and tissues, causing oxidative damage to lipids, proteins, and nucleic acids [4,5]. The up-regulation of motor proteins in KEGG enrichment further suggests that cytoskeletal rearrangements are potentially associated with cellular stress responses and intracellular transport adaptation.

Concurrently with the activation of stress-responsive metabolic pathways, blue light exposure elicited marked transcriptional repression of ribosomal biogenesis and protein synthesis machinery. KEGG analysis identified the ribosome pathway as the most significantly enriched down-regulated category, while GO enrichment revealed suppression of translation, peptide biosynthetic processes, and ribonucleoprotein complex assembly. This inverse transcriptional pattern—up-regulation of catabolic stress responses coupled with down-regulation of anabolic machinery—suggests a metabolic reallocation, wherein cellular resources are diverted from growth-associated biosynthesis toward adaptive stress management. Similar down-regulation of ribosomal protein genes has been documented in *Drosophila* under peroxisomal import stress and in plants responding to biotic and abiotic challenges [50]. The pronounced down-regulation of folate biosynthesis further reinforces this metabolic shift, as folate cofactors are essential for nucleotide synthesis and methylation reactions; their reduced expression likely reflects a generalized slowdown in proliferative and biosynthetic activities under stress conditions [51,52,53,54].

Red light treatment elicited a distinctive transcriptional signature dominated by the up-regulation of lysosomal and autophagy-related pathways. KEGG enrichment analysis identified lysosome, autophagy–animal, and biosynthesis of cofactors as the three significantly up-regulated pathways in RvsCK. Within these pathways, multiple cathepsin family cysteine proteases, including cathepsin B-like cysteine proteinase genes (*CYSP1*, *CPR4*) and pro-cathepsin H gene (*CATH*), exhibited pronounced up-regulation. Cathepsins are lysosomal proteases with pivotal roles in protein turnover, antigen processing, and innate immune defense [55,56,57]. This pattern aligns with stress-responsive cathepsin induction observed in other insects [58]. The significant enrichment of cysteine-type peptidase activity in the GO analysis of RvsCK up-regulated genes further supports the activation of lysosomal proteolytic machinery.

Constant cross-talk between lysosomes and autophagy, in terms of fusion and regulation, underlies steady autophagic flux [59]. Macroautophagy represents a conserved cellular quality control mechanism wherein damaged organelles and protein aggregates are sequestered within autophagosomes and subsequently degraded following lysosomal fusion [60,61]. In *Bombyx mori* Linnaeus, a cathepsin L-like protease has been functionally implicated in anti-microbial autophagy, with its depletion altering Toll and IMD pathway-associated gene expression [62]. The parallel up-regulation of autophagy and lysosomal markers in our red light treatment may reflect activation of similar immune surveillance mechanisms, potentially triggered by light-induced cellular damage or photoreceptor protein turnover.

The red light treatment also induced significant up-regulation of genes associated with lipid metabolism and energy mobilization. Putative fatty acyl-CoA reductase (*FACR1*) and vitellogenin-2 (*VIT2*) were among the top up-regulated DEGs. Vitellogenin serves as a major yolk protein precursor and also functions as an antioxidant and immune modulator in diverse insect species. Its up-regulation under red light may indicate enhanced investment in reproductive reserves or, alternatively, deployment of its antioxidant properties to mitigate light-induced oxidative damage [63,64,65]. Moreover, the up-regulation of juvenile hormone esterase (*JHE*), which catalyzes the hydrolysis of juvenile hormone (JH) and thereby modulates JH titer, suggests altered endocrine signaling under red light conditions. Photoperiod and light quality are well-established regulators of JH metabolism in insects, with JH esterase activity showing photoperiod-dependent fluctuations in diapausing and non-diapausing individuals [66,67,68,69].

Several limitations of this study should be noted. This study employed pooled male and female individuals without sex differentiation, a design that precludes discerning sex-specific transcriptomic responses to monochromatic light perturbation. Future studies should adopt sex-stratified sampling to disentangle wavelength-dependent transcriptional signatures from sexually dimorphic baseline expression. Additionally, this study was conducted at a single light intensity (80 lux). Although this intensity is ecologically relevant to typical urban light pollution scenarios, it is insufficient to resolve dose-dependent transcriptomic thresholds and saturation effects. Future systematic intensity-gradient experiments are needed to establish the quantitative relationship between photon flux density and transcriptional reprogramming, providing a basis for intensity-optimized spectral control strategies in integrated pest management.

The transcriptomic data presented here enable the identification of key genes exhibiting significant differential expression under monochromatic light, which may serve as candidate molecular targets for combined biological and physical control strategies against *D. citri*. Furthermore, this convergence of molecular and behavioral evidence indicates that nighttime illumination in citrus orchards should be managed to minimize blue light pollution and prevent light-induced pest aggregation; concurrently, the documented phototactic sensitivity of *D. citri* to blue light supports deploying blue light trapping devices as a complementary physical control measure.

## 5. Conclusions

In summary, this study systematically characterized the wavelength-dependent transcriptomic reprogramming of *D*. *citri* exposed to nocturnal monochromatic LED illumination. Short-wavelength blue light elicited the most extensive transcriptional response, involving profound metabolic reallocation toward amino acid catabolism, glyoxylate metabolism, and the kynurenine pathway, concomitant with marked suppression of ribosomal biogenesis and protein synthesis machinery, collectively indicative of a coordinated cellular stress adaptation. Long-wavelength red light induced a distinct molecular signature dominated by activation of lysosomal proteolytic and autophagic pathways, alongside enhanced lipid metabolism and energy mobilization. By contrast, mid-wavelength yellow and green light produced relatively modest transcriptional alterations. These results demonstrate that *D*. *citri* exhibits pronounced spectral specificity in its molecular responses to nocturnal monochromatic illumination. Beyond deepening our understanding of the eco-physiological consequences of wavelength-specific light pollution, this study establishes a mechanistic framework for spectrally tailored pest management. Specifically, our findings inform the optimization of nighttime lighting regimes in citrus orchards to minimize blue light exposure and reduce pest aggregation while supporting the deployment of blue light trapping devices as complementary physical control measures. Furthermore, the wavelength-specific differentially expressed genes identified here provide promising candidates for subsequent screening of molecular targets to enable integrated biological–physical control strategies.

## Figures and Tables

**Table 1:** Summary of the transcriptomes in the different treatments in *D. citri*.

Sample	Raw Reads	Clean Reads	Clean Bases (Gb)	Clean Reads Q20 (%)	Clean Reads Q30 (%)	GC (%)	total_map	unique_map	positive_map	negative_map
CK_1	46907172	45146734	6.77	99.17	96.91	36.6	35011751 (77.55%)	33315289 (73.79%)	16703622 (37.0%)	16611667 (36.79%)
CK_2	50223406	47757370	7.16	99.35	97.42	40.35	37206660 (77.91%)	35479986 (74.29%)	17735485 (37.14%)	17744501 (37.16%)
CK_3	41096586	38647732	5.8	99.31	97.16	39.9	29918018 (77.41%)	28541120 (73.85%)	14300889 (37.0%)	14240231 (36.85%)
B_1	43752676	41107156	6.17	99.16	96.9	36.8	31850226 (77.48%)	30320343 (73.76%)	15191541 (36.96%)	15128802 (36.8%)
B_2	48294502	45473698	6.82	99.2	97.06	37.12	35264838 (77.55%)	33282945 (73.19%)	16700980 (36.73%)	16581965 (36.46%)
B_3	47426052	47305844	7.1	99.15	96.82	38.84	37130169 (78.49%)	35313462 (74.65%)	17735739 (37.49%)	17577723 (37.16%)
R_1	44972708	42102276	6.32	99.17	96.9	37.43	32108568 (76.26%)	30411429 (72.23%)	15315690 (36.38%)	15095739 (35.85%)
R_2	47881914	46026312	6.9	99.13	96.76	36.91	35501216 (77.13%)	33911697 (73.68%)	16969171 (36.87%)	16942526 (36.81%)
R_3	46953080	44716786	6.71	99.37	97.44	41.18	35714556 (79.87%)	34099035 (76.26%)	17000734 (38.02%)	17098301 (38.24%)
Y_1	46139834	44402484	6.66	99.37	97.54	35.79	34044483 (76.67%)	32707456 (73.66%)	16476746 (37.11%)	16230710 (36.55%)
Y_2	47219652	45866238	6.88	99.35	97.52	38.48	36031988 (78.56%)	34085977 (74.32%)	17106963 (37.3%)	16979014 (37.02%)
Y_3	48409328	46463922	6.97	99.29	97.24	36.62	35723414 (76.88%)	34224330 (73.66%)	17096809 (36.8%)	17127521 (36.86%)
G_1	44073412	42068826	6.31	99.35	97.45	38.79	32658411 (77.63%)	31276177 (74.35%)	15646770 (37.19%)	15629407 (37.15%)
G_2	49847238	47653938	7.15	99.34	97.37	37.64	36936582 (77.51%)	35125099 (73.71%)	17646587 (37.03%)	17478512 (36.68%)
G_3	49278284	48247394	7.24	98.9	96.48	45.2	39540265 (81.95%)	34914247 (72.37%)	17414480 (36.09%)	17499767 (36.27%)

## Data Availability

The original contributions presented in this study are included in the article/Supplementary Material. Further inquiries can be directed to the corresponding authors.

## Supplementary Materials

The following supporting information can be downloaded at: https://www.mdpi.com/article/10.3390/insects17070735/s1, Table S1: Primers used for RT-qPCR analysis in this study; Tables S2–S5: DEGs in each of the four treatment groups relative to the control group; Tables S6–S9: KEGG pathway enrichment results of DEGs in the four treatment groups relative to the control group; Tables S10–S13: GO enrichment results of DEGs in the four treatment groups relative to the control group.
